# Predictors of long-term outcomes in patients with persistent atrial fibrillation undergoing electrical cardioversion

**DOI:** 10.34172/jcvtr.32913

**Published:** 2024-03-13

**Authors:** Mohammad Reza Dehghani, Navideh Safarzadeh, Akram Shariati, Yousef Rezaei

**Affiliations:** ^1^Department of Cardiology, Seyyed-al-Shohada Heart Center, Urmia University of Medical Sciences, Urmia, Iran; ^2^Heart Valve Disease Research Center, Rajaie Cardiovascular Medical and Research Center, Iran University of Medical Sciences, Tehran, Iran; ^3^Behyan Clinic, Pardis New Town, Tehran, Iran

**Keywords:** Atrial fibrillation, Cardioversion, Prognosis, Coarse atrial fibrillation, Body mass index

## Abstract

**Introduction:**

Cardioversion for atrial fibrillation (AF) is routinely implemented in daily practice; however, it can be associated with the development of recurrent AF. In this study we aimed to evaluate the predictors of AF recurrence after electrical cardioversion, and to compare the outcomes of patients with or without AF recurrence during follow-up.

**Methods:**

Patients with persistent AF were enrolled from March 2015 to September 2018. Patients with recurrent AF within 6 months after the index cardioversion were considered as AF recurrence (AFR) group, and those with normal sinus rhythm were defined as normal sinus rhythm (NSR) group. Thereafter, all patients were followed up for the incidence of adverse events, including death, requiring dialysis, coronary artery intervention/surgeries, cerebrovascular events, heart failure, and recurrent AF beyond 6 months.

**Results:**

Of 129 patients, 11 patients had failed cardioversion and 7 patients lost to follow-up. So, 34 and 77 patients were categorized as the NSR and the AFR groups. During a median follow-up time of 54 (46-75) months, there was a trend for a higher incidence of major adverse events in the AFR group compared to the NSR group (*P*=0.063). Lower body mass index (odds ratio [OR] 0.885, 95% confidence interval [CI] 0.794-0.986, *P*=0.027) and coarse AF before the index cardioversion (OR 3.846, 95% CI 1.189-12.443, *P*=0.025) were the independent predictors of recurrent AF.

**Conclusion:**

In patients with persistent AF undergoing cardioversion, the presence of coarse AF and the lower values of body mass index were found to be associated with the AF recurrence.

## Introduction

 Atrial fibrillation (AF) is the most prevalent cardiac arrhythmia with an increasing rate of incidence due to the aging population.^1^ AF is associated with the increased risk of thromboembolic events and life-threatening events.^2^ It has been estimated that the prevalence of AF will grew during the next 30 years, and it may increase by more than 60% in 2050.^3^

 Several factors impact on the selection of treatment strategies for the management of AF, and rhythm or rate control are implemented in our daily practice. Electrical and chemical cardioversion have been introduced several decades ago as rhythm control strategies,^4^ while catheter ablation has also been emerged as new tool in some of AF patients.^5^ About half of cardioversion procedures failed to maintain sinus rhythm within the first month after the procedure in patients with AF.^6^ The relapse of AF after cardioversion is associated with the increased risk of mortality,^7^ hence several studies tried to find the predictors of AF recurrence after cardioversion so that improve the success rate of rhythm control in these patients. Of note, some of clinical,^8^ electrocardiographic,^9,10^ echocardiographic,^11^ laboratories,^12^ and procedural features^13^ have been found to be associated with the development of AF recurrence after cardioversion.

 In this retrospective study, we sought to determine the predictors of AF recurrence after electrical cardioversion in patients with persistent AF. Moreover, we tried to evaluate the overall prognosis of patients with or without AF recurrence after cardioversion during follow-up period.

## Materials and Methods

###  Study cohort and protocols

 In this retrospective cohort study, a total of 129 consecutive patients with persistent AF who referred to the Electrophysiology Laboratory of our institution, a tertiary center for cardiovascular care in the West Azerbaijan of Iran, were enrolled from March 2015 to September 2018. The inclusion criteria included adult patients with a diagnosis of persistent AF who were scheduled to undergo electrical cardioversion and had at least 6 months follow-up outcomes. Exclusion criteria included patients without follow-up outcomes, patients with failed cardioversion procedure, as well as those with missed data for procedural and clinical features. All patients underwent electrical cardioversion using the biphasic defibrillator device. The protocol for the implementation of electrical shocks included a starting 100 joule with escalating shock energies up to 300 joules every 30-60 seconds in cases without sinus rhythm. In cases with remained sinus rhythm for 15 minutes, those were considered as early successful cardioversion.

 The study protocol was reviewed and approved by the institutional review board of the Urmia University of Medical Sciences, West Azerbaijan Province, and it was conducted according to the Declaration of Helsinki. An informed consent exemption was granted by the institutional review board, Urmia University of Medical Sciences, because all data were retrospectively collected from databases/files. Moreover, the study data were de-identified and utilized for research purposes.

###  Clinical features

 All baseline characteristics were collected from electronic databases and/or stored data sheets. The details of pre-procedural electrocardiogram (ECG) included heart blocks, P wave features in the lead II, pathologic Q wave, QRS axis deviation, Bazett’s corrected QT interval, the presence of fragmented QRS (fQRS) complex, the type of AF (i.e. the greatest amplitude of F wave ≥ 1 mm in lead V1 defined as coarse AF and the F wave amplitude < 1 mm defined as fine AF),^14^ and the ventricular response. The duration of AF was ascertained using the interval between the date of first ECG with AF and the time of cardioversion. The details of echocardiographic examinations were also collected, including systolic function, diastolic function, and any structural findings. The extent of coronary artery disease was also confirmed in cases with documented coronary angiographic evaluation. All drug histories during pre- and post-procedural periods were recorded.

###  Follow-up outcomes

 During follow-up period, all patients were assessed based on the data provided by outpatient clinic visiting data sheets, and telephone interviews. All events were confirmed by reviewing recorded data sheets in our outpatient clinics and hospital wards. The recurrence of AF rhythm during follow-up period was assessed by ECG interpretation. All patients were requested to provide ECGs when they were symptomatic or in a routine care at 6 months intervals. Thereafter, all patients were requested to send their ECGs via cellphone-based softwares or connect us to their local cardiologists so that we could obtain the interpretation of ECGs. Patients with recurrent AF rhythm within 6 months after the index cardioversion were considered as AF recurrence (AFR) group, and those with normal sinus rhythm within 6 months after cardioversion were defined as normal sinus rhythm (NSR) group. During follow-up period, all adverse events were evaluated and the time of events was ascertained. Adverse events comprised of myocardial infarction, cerebrovascular accidents, renal failure, warfarin toxicity, low international normalized ratio (INR) levels, implantable cardioverter defibrillator, hospitalization for acute coronary syndrome, and admission for heart failure, any types of coronary artery interventions, ventricular tachycardia, and death.

###  Anticoagulation therapy

 The presence of valvular or non-valvular AF affected the type of anticoagulation therapy. In those with valvular AF (i.e., ≥ moderate mitral valve stenosis and/or mechanical heart valves), patients were given warfarin to reach an INR level ranging from 2 to 3. For patients with non-valvular AF, rivaroxaban or apixaban with dose adjustments in some cases were applied. Among patients with a history of percutaneous coronary artery intervention, given his/her clinical conditions, those received triple therapy (clopidogrel + aspirin + non-vitamin k antagonist oral anticoagulant/warfarin) for a month and a subsequent dual therapy (clopidogrel + non-vitamin k antagonist oral anticoagulant/warfarin) for 6-12 months and thereafter continued with single therapy (non-vitamin k antagonist oral anticoagulant/warfarin).

###  Statistical analysis

 Categorical variables were reported as number (percentage). Continuous variables were presented as mean ± standard deviation (SD) or median (interquartile range [IQR]). The *t*-test, Mann-Whitney U test, or one-way ANOVA test was applied to compare differences in continuous variables. Chi-squared test was applied to assess the differences between groups regarding the categorical variables. Multivariable logistic regression analysis was used to find the predictors of the recurrence of AF after cardioversion (it was defined as patients with AF recurrence 6 months after the index cardioversion), and odds ratio (OR) with 95% confidence interval (CI) were also reported. Variables with significant effect on the recurrence of AF in prior studies and variables with *P* < 0.1 in the univariate analyses comparing AFR and NSR groups in our study were included in the multivariable regression model. Kaplan-Meier curve was constructed to compare the AFR and NSR groups with regard to the development of major adverse clinical events (MACE; it includes death, requiring dialysis, coronary artery interventions/surgeries, cerebrovascular events, heart failure, and recurrent AF) during follow-up period. The log-rank test was applied to compare both groups. Two-sided p values were calculated. All statistical analyses were performed using STATA software (College Station, TX, USA).

## Results

###  Baseline characteristics

**Figure 1:**
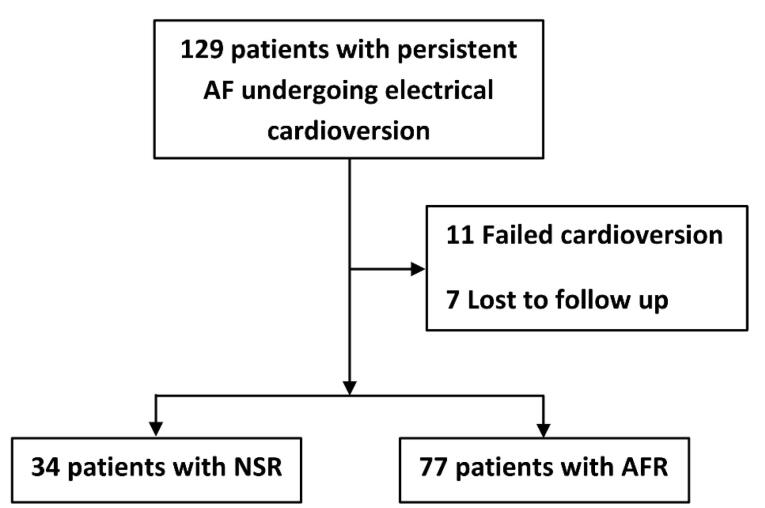
Graph showing included and excluded patients undergoing electrical cardioversion Footnote: AF, atrial fibrillation; AFR, atrial fibrillation recurrence; NSR, normal sinus rhythm

 Of 129 patients, after 6 months follow-up, 34 and 77 patients were categorized as NSR and AFR groups. Of excluded individuals, 11 patients experienced a failed cardioversion, and 7 patients were lost to follow-up during early 6 months after cardioversion (Figure 1). The distributions of age and sex were comparable among excluded patients, NSR, and AFR groups (*P* = 0.441 for sex, and *P* = 0.640 for age). The distribution of conventional cardiovascular risk factors were also comparable among three groups (all *P* > 0.05).

 Patients’ mean age (65 ± 12.3 vs. 64.9 ± 13.7, *P* = 0.961) and sex distribution (50% vs. 50.6% male, *P* = 0.950) were comparable between the study groups, NSR and AFR. About a third of patients in both groups had a history of AF less than 6 months before the index cardioversion (*P* = 0.281). Most of the baseline characteristics were comparable between the study groups (Table 1). Of baseline ECG parameters, the coarse AF rhythm was detected more in the AFR group than the NSR group (88.3% vs. 73.5%, *P* = 0.051). Some other characteristics and ECG features are provided in Supplementary file, Table S1. All echocardiographic findings, mainly including chamber sizes, ventricular functions, and valvular heart diseases were comparable between the study groups (Table 2).

**Table 1:** Baseline characteristics and electrocardiographic features in the study groups

	**NSR** **(n=34)**	**AFR** **(n=77)**	***p*** ** value**
Age, year	65 ± 12.3	64.9 ± 13.7	0.961
Male	17 (50%)	39 (50.6%)	0.950
BMI, kg/m^2^	28.3 ± 5.1	26.8 ± 3.5	0.073
Weight status			0.183
Normal weight	9 (26.5%)	29 (37.7%)	
Overweight	16 (47.1%)	38 (49.4%)	
Obesity	9 (26.5%)	10 (13%)	
Diabetes mellitus	7 (20.6%)	24 (31.2%)	0.252
Hypertension	21 (61.8%)	52 (67.5%)	0.555
Invasive coronary angiography	8 (23.5%)	22 (28.6%)	0.581
Coronary artery interventions^†^	2 (5.9%)	15 (19.5%)	0.067
Cerebrovascular accidents	1 (2.9%)	4 (5.2%)	0.598
Electrocardiographic features			
AF duration			0.281
≤ 6 months	26 (76.5%)	51 (66.2%)	
> 6 months	8 (23.5%)	26 (33.8%)	
AF rhythm type			0.051
Fine	9 (26.5%)	9 (11.7%)	
Coarse	25 (73.5%)	68 (88.3%)	
HR for AF rhythm, bpm	113.8 ± 29.7	116 ± 31.7	0.738
HR for sinus rhythm, bpm	63.1 ± 7.5	63 ± 7.5	0.981
fQRS in any leads	6 (17.6%)	18 (23.4%)	0.499
Corrected QT interval^‡^	405 (380-430)	420 (390-430)	0.244
RVH^§^	2 (5.9%)	3 (3.9%)	0.642
LVH^§^	19 (55.9%)	45 (58.4%)	0.801
Drug histories before cardioversion			
β-blockers	12 (35.3%)	41 (53.2%)	0.081
Statins	18 (52.9%)	43 (55.8%)	0.777
Diltiazem	1 (2.9%)	4 (5.2%)	0.598
Verapamil	2 (5.9%)	4 (5.2%)	0.883
Digoxin	4 (11.8%)	12 (15.6%)	0.597
Amiodarone	3 (8.8%)	6 (7.8%)	0.854
Flecainide	0	3 (3.9%)	0.243
NLR	1.89 (1.34-2.40)	1.90 (1.26-2.70)	0.639

All data are presented as number (%), mean ± SD, and median (IQR) ^†^ It includes percutaneous coronary interventions and coronary artery bypass graft surgeries ^‡^It has been corrected using the Bazett’s formula ^§^Electrocardiographic changes indicating ventricular hypertrophies AF, atrial fibrillation; AFR, atrial fibrillation recurrence; BMI, body mass index; fQRS, fragmented QRS complex; HR, heart rhythm; LVH, left ventricular hypertrophy; NLR, neutrophil to lymphocyte ratio; NSR, normal sinus rhythm; RVH, right ventricular hypertrophy *P* value< 0.05 statistically significant.

**Table 2:** Baseline echocardiographic features of patients in the study groups

	**NSR** **n=34**	**AFR** **n=77**	***p*** ** value**
LVEF, %	45 (35-50)	50 (35-55)	0.700
LVESD, cm	3.4 (3-3.9)	3.6 (3.2-4)	0.323
LVEDD, cm	4.9 (4.6-5.3)	4.9 (4.5-5.3)	0.558
LA area, cm^2^	21 (18-25)	21 (18-25)	0.419
RVEF			0.762
Normal	28 (82.4%)	59 (76.6%)	
Mild dysfunction	2 (5.9%)	8 (10.4%)	
Moderate dysfunction	1 (2.9%)	1 (1.3%)	
Severe dysfunction	3 (8.8%)	9 (11.7%)	
RV size, cm	2.9 (2.7-3.2)	2.8 (2.7-3.1)	0.548
RA area, cm^2^	17.5 (15-21)	17 (14-20)	0.722
VHD			
Normal or mild changes	8 (23.5%)	19 (24.7%)	0.897
Moderate/Severe MR	18 (52.9%)	40 (51.9%)	0.923
Moderate/Severe TR	20 (58.8%)	40 (51.9%)	0.503
Moderate/Severe AI	5 (14.7%)	14 (18.2%)	0.654
Moderate/Severe PI	2 (5.9%)	4 (5.2%)	0.883
Other VHDs	2 (5.9%)	7 (9.1%)	0.568
Moderate LVH	3 (8.8%)	6 (7.8%)	0.854
Severe LVH	1 (2.9%)	9 (11.7%)	0.138
sPAP, mm HG	15 (15-30)	15 (15-30)	0.836
Pericardial effusion	1 (2.9%)	1 (1.3%)	0.549

All data are presented as number (%), mean ± SD, and median (IQR) AFR, atrial fibrillation recurrence; AI, aortic valve insufficiency; LA, left atrial; LVEDD, left ventricular end diastolic diameter; LVEF, left ventricular ejection fraction; LVESD, left ventricular end systolic diameter; LVH, left ventricular hypertrophy; MR, mitral valve regurgitation; PI, pulmonary valve insufficiency; RA, right atrial; RV, right ventricular; RVEF, right ventricular ejection fraction; NSR, normal sinus rhythm; sPAP; systolic pulmonary arterial pressure; TR, tricuspid valve regurgitation; VHD, valvular heart disease. *P* value< 0.05 statistically significant.

###  Cardioversion features

 During the index cardioversion, the most commonly used drug was amiodarone (96.4% of patients, Table 3). The use of drugs during the index cardioversion was comparable between both groups, except for a combination of amiodarone + diltiazem + digoxin, which was significantly higher in the NSR group compared to the AFR group (8.8% vs. 1.3%, *P* = 0.050). The majority of patients received only 1 shock during the index cardioversion (79.4% and 74% in the NSR and AFR groups, respectively). The amount of joules during the index cardioversion was comparable between both groups (150 [150-200] vs. 150 [150-325], *P* = 0.622). The minimum and maximum amounts of joules in the applied cardioversion shocks upon first attempt were 50 (in 6 patients, 5.4%) and 150 (in 68 patients, 61.3%) joules, respectively. All drugs upon discharge after the index cardioversion included β-blockers, digoxin, amiodarone, verapamil, diltiazem flecainide, and statins. The distribution of drugs upon discharge were comparable between the study groups (all *P* > 0.05; those values are not mentioned within the tables).

**Table 3:** Cardioversion features in the study groups

	**NSR** **n=34**	**AFR** **n=77**	***p*** ** value**
Drugs during cardioversion			
Amiodarone + β-blocker	15 (44.1%)	35 (45.5%)	0.896
Amiodarone + Verapamil	2 (5.9%)	7 (9.1%)	0.568
β-blocker + Verapamil	0	2 (2.6%)	0.343
β-blocker + Diltiazem	0	1 (1.3%)	0.504
Amiodarone + Diltiazem	1 (2.9%)	3 (3.9%)	0.803
Amiodarone + Diltiazem + Digoxin	3 (8.8%)	1 (1.3%)	0.050
Amiodarone + Flecainide	0	1 (1.3%)	0.504
Flecainide + Verapamil	0	1 (1.3%)	0.504
Amiodarone + β-blocker + Digoxin	15 (44.1%)	30 (39%)	0.610
Number of shocks			0.269
1 shock	27 (79.4%)	57 (74%)	
2 shocks	6 (17.6%)	8 (10.4%)	
3 shocks	1 (2.9%)	12 (15.6%)	
Amount of cardioversion joules, j	150 (150-200)	150 (150-325)	0.622

All data are presented as number (%) and median (IQR) AFR, atrial fibrillation recurrence; NSR, normal sinus rhythm. *P* value< 0.05 statistically significant.

###  Outcomes after discharge

**Figure 2:**
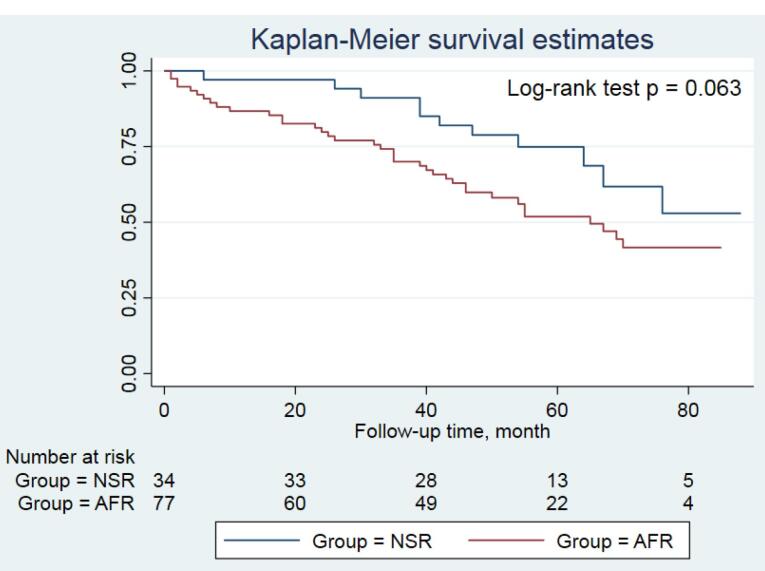
Kaplan-Meier curve comparing the study groups regarding the freedom from MACE during follow-up period Footnote: AFR, atrial fibrillation recurrence; NSR, normal sinus rhythm

**Figure 3:**
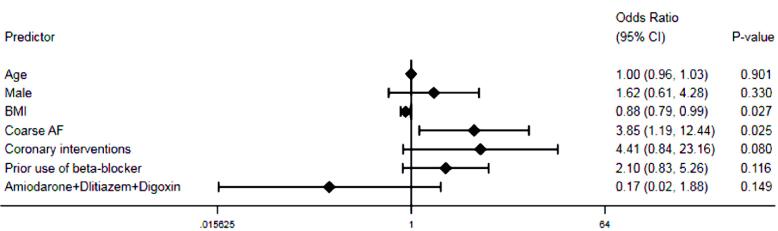
Multivariable logistic regression analysis depicting the predictors of AF recurrence during follow-up period Footnote: AF, atrial fibrillation; BMI, body mass index; CI, confidence interval; NSR, normal sinus rhythm

 All patients were followed up for a median of 54 (46-75) months. Amiodarone side effects (i.e. edema, hypothyroidism, and corneal deposits which required the discontinuation of drug) were comparable between both groups; those were occurred in 3 patients. Warfarin intoxication and low INR were also detected in 1 (2.9%) and 8 (10.4%) patients in the NSR and the AFR groups, respectively (*P* = 0.185). The number of patients undergoing coronary interventions was higher in the AFR group than those in the NSR group (19.5% vs. 5.9%, *P* = 0.067). The number of individuals admitted for the recurrence of AF during follow-up period after first 6 months was significantly higher in the AFR group compared to the NSR group (5.9% vs. 29.9%, *P* = 0.005). Other events during follow-up period were comparable between both groups, summarized in Table 4. The Kaplan-Meier curve showed a trend for a higher incidence of MACE at follow-up period in the AFR group compared to the NSR group, indicating worse outcomes for patients experiencing the recurrence of AF (*P* = 0.063, Figure 2). Moreover, based on the multivariable logistic regression analysis, lower body mass index (OR 0.885, 95% CI 0.794-0.986, *P* = 0.027) and the coarse AF (OR 3.846, 95% CI 1.189-12.443, *P* = 0.025) were the independent predictors of the recurrent AF (Figure 3).

**Table 4:** Clinical outcomes during follow-up period in the study groups

	**NSR** **n=34**	**AFR** **n=77**	***p*** ** value**
Re-admission reasons at follow-up			
Warfarin intoxication	1 (2.9%)	8 (10.4%)	0.185
Low INR	0 (0%)	2 (2.6%)	0.343
Hypertension crisis	1 (2.9%)	0 (0%)	0.131
ACS	1 (2.9%)	3 (3.9%)	0.803
Coronary interventions^†^	2 (5.9%)	15 (19.5%)	0.067
Holter monitoring	1 (2.9%)	2 (2.6%)	0.918
Ventricular tachycardia	0 (0%)	2 (2.6%)	0.343
AF^‡^	2 (5.9%)	23 (29.9%)	0.005
CD	1 (2.9%)	5 (6.5%)	0.446
CVE	0 (0%)	1 (1.3%)	0.504
Heart failure	3 (8.8%)	8 (10.4%)	0.799
Requiring renal dialysis	0 (0%)	2 (2.6%)	0.343
Death	7 (20.6%)	16 (20.8%)	0.982
MACE^¶^	11 (32.4%)	37 (48.1%)	0.124

All data are presented as number (%) ACS, acute coronary syndrome; AF, atrial fibrillation; AFR, atrial fibrillation recurrence; CD, cardiac defibrillation; CV, cardiovascular; CVE, cerebrovascular events; INR, international normalized ratio; MACE, major adverse clinical events; NSR, normal sinus rhythm ^†^Coronary interventions included percutaneous coronary interventions and coronary artery bypass graft surgeries ^‡^Re-admission for AF rhythm after 6 months from the date of study entrance ^¶^MACE included death, requiring dialysis, coronary artery intervention/surgeries, cerebrovascular events, heart failure, and recurrent AF beyond 6 months during follow-up period. *P* value< 0.05 statistically significant.

## Discussion

 In this retrospective single-center study we found that among patients with persistent AF undergoing electrical cardioversion, approximately 60% of individuals experienced the recurrence of AF during 6 months after the index cardioversion. During the follow-up, patients with recurrent AF experienced more ischemic heart disease events requiring coronary artery interventions/surgeries compared to those without the recurrent AF. The rate of freedom from MACE during follow-up period was higher in the NSR compared to the AFR group. Moreover, according to the multivariable analysis, the presence of coarse AF and a lower body mass index were associated with the recurrent AF among the study population.

 Several studies have evaluated the predictors of outcomes among patients with persistent AF who underwent cardioversion. Ecker et al.^15^ reviewed literatures and provided an evidence for mostly reported predictors of outcomes of cardioversion for AF. Obesity has been demonstrated as a main factor for recurrence after cardioversion, while we found that the lower values of body mass index was associated with increased odds of AF recurrence. Obesity is a known risk factor or the development of AF; however, some studies found that underweight and normal weight individuals who were diagnosed with AF had worse prognosis than obese counterparts in two different population-based studies.^16,17^ There are few reports focusing on the weight effect on the outcomes of patients undergoing the cardioversion for AF. We think that further studies are deemed indicated for evaluating the effect of weight on the outcomes of cardioversion for AF. Female gender has also been found to be a predictor of AF recurrence after cardioversion,^8^ but we did not find any effect of sex on AF recurrence in our population.

 Electrocardiographic features can be associated with the prognosis of cardioversion. Eren and colleagues revealed that the presence of fragmented QRS was an independent predictor of AF recurrence (hazard ratio 9.670, 95% CI 4.714-19.837, *P* < 0.001) after successful cardioversion in patients with persistent non-valvular AF.^18^ In a cohort of 502 patients with persistent AF referred for cardioversion, after 1 year follow-up, the rate of sinus rhythm maintenance was found to be 32%. The main predictors of sinus rhythm maintenance included the use of anti-arrhythmic agents. They also found that the addition of electrocardiographic parameters improved the prediction accuracy from 0.62, using only clinical parameters, to 0.67, combining clinical and electrocardiographic parameters.^10^ Moreover, the duration of AF was also associated with the AF recurrence so that the shorter duration of AF was associated with the maintenance of sinus rhythm during 1-year follow-up period^4^ and longer duration of AF predicted AF recurrence during a 5-year follow-up period.^19^ In our population, we demonstrated that the presence of coarse AF rhythm was a main predictor of AF recurrence during follow-up period (OR 3.85, 95% CI 1.19-12.44, *P* = 0.025). However, none of pre- and post-cardioversion anti-arrhythmic agents and the duration of AF were associated with the outcomes of cardioversion. In prior reports it has been demonstrated that the coarse AF was associated with a higher success rate of cardioversion.^20-22^ Of note, all these reports had short- to mid-term follow-up periods (up to 1 year), while we followed up patients with a median of 54 months, which may in part lead to different finding. Moreover, we showed that the thinner individuals were also associated with the presence of successful cardioversion. We think that the presence of some confounders can significantly affect the relationship between cardioversion outcomes and baseline features. In addition, there are evidences indicating the association between the duration of AF and changing coarse AF to fine AF. On the other hand, there is evidence showing the higher probability of becoming sinus rhythm and remaining in sinus rhythm in coarse AF; however, it was not associated with the duration of AF.^22^ One of the reasons for changing coarse AF to fine AF includes atrial remodeling. The duration of AF has been found as a contributor of atrial remodeling, but rate-induced intracellular calcium overload has also been shown to cause atrial remodeling independent of time, so that the atrial remodeling could develop in a short time course too.^23^ No conclusion about the mechanistic relationship can be drawn from our study. Further studies are deemed indicated to evaluate such associations.

 In our population, there were no significant differences between both groups with regard to the echocardiographic parameters. All structural features in echocardiographic evaluations, including valvular heart diseases, chamber sizes, and ventricular functions were comparable among patients with or without AF recurrence. On the other hand, prior studies revealed that larger left atrium and worse left atrial systolic^24^ as well as left ventricular diastolic dysfunction^25^ predicted the recurrence of AF after cardioversion. Moreover, some of cardioversion features were associated with AF recurrences so that high energy shocks and prior history of cardioversion predicted AF recurrence 1 year after index cardioversion. In contrast, in our population the number of shocks and the amount of shock energies were comparable between both the AFR and NSR groups. Further studies with longer duration of follow-up may help us with finding the predictors of AF recurrence in patients with persistent AF undergoing cardioversion.

 This study has some limitations need to be addressed in future investigations. First, the small sample size prevents us from further analysis to evaluate the association between more features predicting the outcomes of cardioversion in patients with persistent AF. Second, we did not implement holter monitoring for the detection of rhythm abnormalities during follow-up period and the diagnosis of AF recurrence was based on 12-lead electrocardiogram upon follow-up visit. This shortcoming can be observed in the majority of studies about the rate control, and it resulted in the underestimation of AF detection, because we diagnosed clinical recurrence and/or apparent AF during follow-up period. The conduction of large-scale studies focusing on electrocardiographic, echocardiographic, and cardioversion features can provide us valuable information about the proper predictors of AF recurrence in patients undergoing cardioversion.

## Conclusion

 Our findings based on a single-institutional cohort of patients with persistent AF who followed up for a long-term period contributed to identifying predictors of recurrent AF after electrical cardioversion. We revealed that the presence of coarse AF and the lower values of body mass index independently predicted the recurrence of AF after cardioversion. This study also showed that the higher incidence of MACE during follow-up period among patients with recurrent AF underscores the need for the implementation of more strict management of patients with failed cardioversion.

## Acknowledgements

 We warmly thank to the nursing and documentary departments in Seyyed-al-Shohada Heart Center, whose members helped us with the data collection.

## Competing Interests

 The authors have no relevant financial or non-financial interests to disclose.

## Ethical Approval

 The study protocol was approved by the local ethics committee of UMSU under the identification number of “IR.UMSU.REC.1398.102”.

## Funding

 None.

## Supplementary Files

Supplementary File contains Table S1.
