# Supplementary material for: Predictors of long-term outcomes in patients with persistent atrial fibrillation undergoing electrical cardioversion
Source: J Cardiovasc Thorac Res. 2024 Mar 13;16(1):21–7. doi: 10.34172/jcvtr.32913 (PMC10997977; doi:10.34172/jcvtr.32913)
Supplement: Supplementary file 1 — Supplementary File contains Table S1. [file jcvtr-16-21-s001.pdf]

J Cardiovasc Thorac Res, 2024, 16(1), 21-S1
doi: 10.34172/jcvtr.2024.32913
http://jcvtr.tbzmed.ac.ir

Table S1. Other characteristics and electrocardiographic findings in the study groups

	NSR (n = 34)	AFR (n = 77)	p value
Number of risk factors†			0.391
None	6 (17.6%)	12 (15.6%)	
At least 1	15 (44.1%)	25 (32.5%)	
>1	13 (38.2%)	40 (51.9%)	
Normal P wave height in lead II	34 (100%)	77 (100%)	1
P wave time in lead II, s	0.4 (0.4-0.8)	0.4 (0.4-0.8)	0.608
Normal PR interval in lead II	31 (91.2%)	73 (94.8%)	0.670
Pathologic Q wave	1 (2.9%)	3 (3.9%)	0.803
QRS complex duration, s	0.1 (0.08-0.12)	0.1 (0.08-0.12)	0.691
QRS axis			0.192
Normal axis	34 (100%)	70 (90.9%)	
Left axis deviation	0	1 (1.3%)	
Right axis deviation	0	6 (7.8%)	
Heart blocks			0.221
None	33 (97.1%)	67 (87%)	
Atrioventricular block	1 (2.9%)	5 (6.5%)	
LAHB	0	5 (6.5%)	

All data are presented as number (%), mean ± SD, and median (IQR)

† Number of cardiovascular risk factors including diabetes mellitus, cerebrovascular accidents, invasive coronary angiography, hypertension, coronary artery interventions, and obesity

AFR, atrial fibrillation recurrence; LAHB, left anterior hemiblock; NSR, normal sinus rhythm
